# Positive Mental Attitude Associated with Lower 35-Year Mortality: The Leisure World Cohort Study

**DOI:** 10.1155/2018/2126368

**Published:** 2018-11-25

**Authors:** Annlia Paganini-Hill, Claudia H. Kawas, María M. Corrada

**Affiliations:** ^1^Department of Neurology, School of Medicine, University of California, Irvine, California, USA; ^2^Department of Neurobiology & Behavior, School of Biological Sciences, University of California, Irvine, California, USA; ^3^Institute for Memory Impairments and Neurological Disorders, University of California, Irvine, California, USA; ^4^Department of Epidemiology, School of Medicine, University of California, Irvine, California, USA

## Abstract

**Background:**

Although emerging research has suggested that “positive psychological well-being” is associated with better health outcomes, studies of long-term health and mortality in the elderly are limited. This study assessed the relationship of mental attitude and mortality in older adults followed up for 35 years.

**Methods:**

In the 1980s, the Leisure World Cohort Study recruited residents of a California retirement community to a prospective cohort study of health promotion and disease prevention. Participants completed a postal survey including seven positively worded items from the Zung self-rating depression scale. Age-adjusted and multivariable-adjusted (for lifestyle behaviors and disease conditions) hazard ratios (HRs) for death were calculated using Cox regression for 8682 women and 4992 men (median age at entry, 74 years). During follow-up (1981–2016), 13,405 participants died (median age at death, 88 years).

**Results:**

In both women and men, HRs for death were significantly related to mental attitude with increasing risk with decreasing positive responses for total attitude and the seven individual items. The multivariable-adjusted HR (95% CI) for death for individuals in the lowest vs. highest quarter of total attitude was 1.24 (1.16, 1.32) for women and 1.30 (1.19, 1.41) for men. Some attenuation in the observed associations occurred after adjustment for potential confounders and after elimination of the first five years of follow-up.

**Conclusions:**

Our study suggests that persons with negative attitude have an increased risk of death even after many years of follow-up. Research into strategies to improve mental outlook may help improve the quantity as well as the quality of life.

## 1. Introduction

The number of elderly adults continues to increase as does the need for them to lead lives in good mental and physical condition. Although “positive mental attitude,” “psychological well-being,” “life satisfaction,” and “happiness” are vague concepts, emerging research suggests that they are associated with better health outcomes [1–5] while their opposites “negative mental attitude,” “life dissatisfaction,” “pessimism,” and “depression and depressive symptoms” are associated with poorer outcomes [6–9].

Studies of mental attitude and long-term health (including mortality) in the elderly are limited by sample size and length of follow-up. Four prospective cohort studies of all-cause mortality in the elderly have included more than 10,000 participants [10–13], and only one of these included both sexes [10]. Follow-up has been greater than 10 years in only three studies [10, 14, 15]. Thus, little is known about whether positive mental attitude has any predictive value in old age over a long-time span.

In 1981, we undertook a prospective cohort study of nearly 14,000 elderly women and men in a California retirement community with the aim of studying factors associated with longevity and successful aging. We report here the results of positive mental attitude (seven items from the Zung self-rating depression scale [16]) on all-cause mortality with 35 years of follow-up. To determine whether mental attitude is associated with mortality independently of known predictors of mortality, we adjusted for medical history (hypertension, angina, heart attack, stroke, cancer, diabetes, and arthritis), body mass index, and lifestyle practices (smoking, alcohol consumption, caffeine intake, and exercise). In addition, the large size of our cohort allowed us to analyze the two sexes separately. As those with health problems may have a poorer mental attitude in the time immediately preceding death, the elimination of early deaths permitted us to determine the long-term effect of mental attitude. Thus, our study adds to the existing knowledge on well-being and mortality in the aged.

## 2. Methods

### 2.1. Participants and Vital Status

The Leisure World Cohort Study was established in the early 1980s when 13,978 residents (8,877 women and 5,101 men) of a California retirement community (Leisure World Laguna Hills) completed a postal health survey. Recruitment procedures have been previously described [17, 18]. Briefly, residents were recruited in 4 waves: those who owned homes in Leisure World on June 1, 1981, and new residents who had moved into the community and were living there on June 1, 1982, June 1, 1983, and October 1, 1985. Recruitment included mailing the survey with simultaneous advertisements describing the study in the local community newspaper, second and third mailings to nonrespondents, and then telephoning nonrespondents (if a local phone number was listed). The baseline survey asked for information on demographic characteristics (sex, marital status, height, and weight), basic medical history (high blood pressure, heart attack, angina, stroke, diabetes, rheumatoid arthritis, glaucoma, fractures, and cancer), and several personal habits (smoking, alcohol consumption, caffeine intake, vitamin supplement use, and physical activity). The Leisure World population and the cohort are predominantly Caucasian, well-educated, and upper-middle class. Vital status of cohort members was determined by periodic resurvey, annual mailings, search of death indexes, and ascertainment of death certificates. Participants were followed to death or December, 31, 2016, whichever came first. To date, 25 cohort members have been lost to follow-up, including 5 who moved out of the country.

This study was approved by the Institutional Review Boards of the University of Southern California and the University of California, Irvine.

### 2.2. Mental Attitude Variables

The survey included only the seven positively worded items from the 20-item Zung self-rating depression scale [16]; the other 13 items were not asked. Participants were asked to read the seven items (Table 1) and to indicate “how much of the time the statement describes how you have been feeling during the past week.” Possible responses were “none or a little of the time, some of the time, a good part of the time, most or all of the time” and were scored 1 through 4, respectively. A total attitude score (7–28) was calculated by summing the seven responses. A score of 7 indicates that the subject selected “none or a little of the time” as a response to all items and represents the poorest possible mental outlook. A score of 28 indicates that all responses were “most or all of the time” and represents the most positive mental outlook. To account for items with missing responses, we calculated the total attitude score in three ways: (1) calculating the total score only for participants without missing responses, (2) assigning a nonresponse item the lowest score of 1 and including it in the total attitude score, and (3) assigning a nonresponse item the highest score of 4 and including it in the total attitude score. For analyses of individual items, those with missing responses were excluded from the analyses.

### 2.3. Potential Confounding Variables

Several factors asked on the same survey with the mental attitude variables were previously found to be related to mortality in this cohort. We included these in analyses as potential confounders. *Medical history* was elicited in response to a question “Has a doctor ever told you that you have any of these conditions?” *Smoking* refers to cigarette use [19]. We estimated *daily caffeine intake* by summing the frequency of consumption of each beverage and chocolate multiplied by its average caffeine content (milligrams/standard unit) as 115, 3, 50, 50, and 6 for regular coffee, decaffeinated coffee, tea, cola soft drinks, and chocolate, respectively [20]. Consumption of *alcoholic beverages* was asked separately for wine, beer, and hard liquor and combined into number of alcoholic drinks per day [21]. *Body mass index* (BMI) (weight (kilograms)/height^2^ (meters)) was calculated based on self-reported height and weight at baseline and categorized according to federal guidelines: underweight (<18.5), normal weight (18.5–24.9), overweight (25–29.9), and obese (30+) [22]. *Exercise* included active outdoor activities (e.g., swimming, biking, jogging, tennis, and vigorous walking) and active indoor activities (e.g., exercising and dancing). The time spent per day in active activities was calculated by summing the reported times spent in active outdoor and active indoor activities [23]. Previous reports present details of data collection [17–24].

### 2.4. Statistical Analysis

Differences between women and men were tested using *t*-tests for continuous variables and chi-squared tests for categorical variables. Hazard ratios (HRs) for the association between mental attitude and mortality were calculated separately for women and men using Cox regression analysis [25] with age as the time scale. Participants contributed person-years from age at baseline survey (delayed entry) to age at death or December 31, 2016, whichever occurred first. Total attitude (continuous and quartile categories) as well as the seven individual items were analyzed as independent variables. The reference category for the HRs was “good mental attitude,” i.e., response of “most or all of the time” for the seven individual items and a total attitude score of 27-28. To control for potential confounders, we performed analysis adjusting for factors previously found to be related to mortality in this cohort: smoking (never, past, and current), alcohol intake (0, ≤1, 2-3, and 4+ drinks/day), caffeine (<50, 50–99, 100–199, 200–399, and 400+ mg/day), exercise (0, ¼, ½, ¾-1¾, and 2+ hour/day), BMI (underweight, normal, overweight, and obese), and histories (yes/no) of hypertension, angina, heart attack, stroke, diabetes, rheumatoid arthritis, and cancer [19–23]. To account for the possibility that recent disease development may have altered attitude as well as be related to mortality, we repeated the analyses after excluding the first five years of follow-up. We performed sensitivity analyses to explore how results would change if the total attitude score on the Zung scale was computed with missing items assigned to either the lowest value (1 = none or little of the time) or alternatively to the highest value (4 = all or most of the time). Statistical analyses were performed using SAS® version 9.4 (SAS Institute Inc., Cary, NC). All tests were two-sided, and no adjustment in the *p*-values was made for multiple comparisons.

## 3. Results

After excluding 89 subjects with missing information on all seven attitude items and an additional 215 with missing information on potential confounding variables, data on 8682 women and 4992 men were analyzed. At study entry, the participants ranged in age from 44 to 101 years (median, 74 years). By December 31, 2016, 13,405 (98%) had died at ages 59 to 110 years (median, 88 years).

Table 1 presents the responses to the seven individual attitude questions. While 13,017 (95%) participants answered all seven questions, 443 did not answer one, 123 two, 28 three, 20 four, 9 five, and 34 six. The distributions of responses differed among the seven statements. The statement “I find it easy to do the things I used to do” had the least number of “most or all of the time” responses (31%) and far fewer than the other statements (53–69%).

Table 2 gives selected characteristics of the participants by sex. Men were on average older than women at study entry (74 vs. 73 years), and a smaller proportion were alive at the end of follow-up (0.9% vs. 2.7%). Men also had on average a greater BMI, exercised more, and consumed more alcohol and caffeine than women, but a smaller proportion never smoked. More men had a history of angina, heart attack, stroke, and diabetes, while more women had high blood pressure, cancer, and rheumatoid arthritis. Women were more likely to have a higher attitude score than men (score ≥25: 52% vs. 50%). All differences were statistically significant (*p* < 0.02).

**Figure 1:**
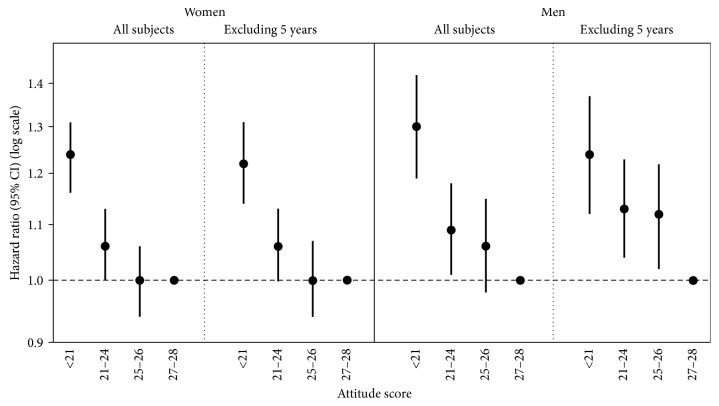
Attitude score and hazard ratios of death: the Leisure World Cohort Study, 1981–2016. Note: data shown are from analyses of total attitude score as quartile categories excluding persons with missing items. HRs and 95% confidence intervals derived from Cox regression analysis model 2, which adjusts for age (as the time scale), smoking, body mass index, exercise, alcohol intake, caffeine consumption, and histories of hypertension, angina, heart attack, stroke, diabetes, rheumatoid arthritis, and cancer.

HRs of mortality for the seven individual attitude items and for the total attitude score are shown in Table 3 for women and in Table 4 for men. Analysis of total attitude score by quartiles showed higher mortality with lower scores (more negative attitude) in both women and men (Figure 1). In the model fully adjusted for age and potential confounders, women with scores of <21 had 24% greater risk of death and men had a 30% greater risk of death compared with persons with scores of 27-28; these risks were 22% and 24% after exclusion of the first five years of follow-up including 854 deaths in women and 1102 deaths in men. As a continuous variable, risk of death significantly increased 2% (women) and 4% (men) for each unit decrease in the total attitude score. In sensitivity analyses that calculated the total score by assigning either the lowest value (1 = none or little of the time) or the highest value (4 = all or most of the time) to the missing items, the results were very similar to those where individuals with missing items were excluded when computing the total score (Tables 3 and 4).

The seven attitude items similarly showed a higher risk of death with increasing level of negative attitude. Adjustment for potential confounders resulted in modest attenuation of risks (less than 20%), and the HRs for poor mental attitude (responses of “none or little of the time” and “some of the time”) remained statistically significant for all items except “I find it easy to make decisions” in women. For all seven items, the HRs for the poorest mental attitude (response of “none or little of the time”) were larger in men than women (about 20% or more except for “My mind is clear as it used to be”). With elimination of the first five years of follow-up, the age-adjusted HRs for women changed by less than 10% for all items except “My mind is as clear as it used to be,” where the HR for the poorest mental attitude (response of “none or little of the time”) was reduced from 1.43 to 1.14. The multivariable-adjusted HRs showed smaller reductions, and the HRs for the poorest mental attitude (response of “none or a little of the time”) for three of the seven items (“mind is clear,” “easy to make decisions,” and “feel useful and needed”) were no longer statistically significant. For men with the elimination of the first five years of follow-up, the age-adjusted HRs for the poorest mental attitude (response of “none or a little of the time”) for all seven items were reduced 10–15% but remained statistically significant; the HRs for other response categories changed by less than 10%. The multivariable-adjusted HRs showed smaller reductions, and HRs for the poorest mental attitude remained statistically significant for all seven items.

## 4. Discussion

We found modest increased risks of death with poor mental attitude that persisted after adjustment for potential confounders including disease history and lifestyle practices. The observed risks, especially for the most negative attitudes, were attenuated after eliminating the first five years of follow-up suggesting that diseases leading to early death might be the cause of some of the negativity. The associations found after adjustment and elimination of the first five years of follow-up may thus be more accurate from a perspective of a causal relationship. Although mental attitude is associated with lifestyle practices and disease states which are also related with mortality, a poor attitude appears to confer additional risk of death.

Those with a positive mental attitude or psychological well-being differ from those with a more negative outlook on a number of health processes—healthier lipid profile, lower levels of inflammatory markers, higher levels of serum antioxidants, better immune responses, and healthier autonomic function [26]—and on a number of healthier behaviors—increased physical activity, nonsmoking, healthier diets, and higher quality of sleep [27]. These may partly serve as mechanisms for the association of mental attitude with mortality.

Previous prospective population-based studies have evaluated mental attitude and all-cause mortality in older population groups [10–15, 28–35]. However, follow-up has generally been less than 10 years, the cohort size less than 10,000, or the study has included only one sex.  Table 5 summarizes these studies. Similar to our study, all previous studies except two [12, 28] found that persons with the most positive attitude (measured in different ways) had the lowest risk of death, and that the risk was attenuated after adjustment of health conditions and lifestyle factors. An inverse dose-response relationship (decreased risk of mortality with increasing levels of positive mental attitude) was seen in both women and men and in studies conducted in diverse countries (USA, Canada, England, the Netherlands, Spain, Sweden, Finland, and China).

As in previous studies, risk of death was attenuated after adjustment of health conditions and lifestyle factors associated with both mental attitude and survival. To reduce concerns that recent changes in mental attitude may be due to underlying illness, we analyzed the data excluding participants who died within five years of study baseline. Likewise, early deaths were eliminated in the Nurses' Health Study (within two years) [11] and the Million Women Study (within five years) [12] to reduce these concerns about reverse causality. However, mental attitude may alter the time course of disease processes and influence health behaviors directly, so the possibility of overadjustment exists and we may be adjusting for the effect of intermediate factors in the causal pathway.

Although some studies have evaluated the association of the Zung self-rating depression scale with mortality [36–38], only those reporting results on the individual items can be directly compared to our study. Takeida and colleagues analyzed the Zung scale as a predictor of death in a Japanese cohort of 2,166 aged 60–74 years and followed five years [39]. Of the seven items included in our study, they found six significantly related to mortality. Those items and their relative risks (RR) were “I find it easy to do the things I used to” (RR = 3.84), “My life is pretty full” (RR = 2.39), “I find it easy to make decisions” (RR = 2.27), “My mind is as clear as it used to be” (RR = 2.05), “I feel hopeful about the future” (RR = 1.67), and “I feel that I am useful and needed” (RR = 1.44). The item “I still enjoy the things I used to do” (RR = 1.35) was not related to mortality. In our older cohort, all seven items were related to mortality. That the individual items we used had a high predictive value of risk of death indicates that refusing to answer any single question does not invalidate the use of the questionnaire nor does the use of the seven rather than the 20 items of the Zung scale.

Several strengths and limitations of this study must be considered. Our data on mental attitude were self-reported using a mailed questionnaire. The seven items included in our survey were extracted from the Zung self-rating depression scale, but we did not ask the other 13 items. Thus, we are not able to compare our results with studies reporting the full Zung scale. Similar to other studies, we previously found our mental attitude score to be related to suicide in the first five years of follow-up [40]. However, conclusions regarding attitude are limited by the crudeness of measurement. Although assessing the true level of attitude is difficult, self-reported rating is suitable for ranking of individuals.

Our large cohort size allowed us to analyze men and women separately. We did this for comparison with other studies reporting sex-specific results [11, 13, 30], because of differences between men and women on baseline characteristics and mortality rates, and due to the possibility of differential response on attitude between men and women. Chang and coworkers showed significant sex differences in reporting of psychological outcomes with more women reporting negative outcomes [41]. This was also seen in several of the prospective studies of attitude and mortality [29, 31], but they did not report sex-specific HRs for death. Differential reporting may help explain the lower HRs for negative attitudes we observed in women compared with men. The cohort reported here is elderly, white, and in the upper middle socioeconomic stratum. Therefore, our results may not be generalizable to other populations.

Our study has the advantages of a prospective design, large size, long and essentially complete follow-up, and the capability to control for numerous potential confounding factors. Still our investigation is an observational study, not a randomized trial. It does, however, suggest that confounders account for only a portion of the associations between mental attitude and risk of death. However, residual confounding due to suboptimal variables or to unmeasured variables remain possibilities.

## 5. Conclusions

Results in this large elderly cohort with long follow-up are consistent with a modest relationship between mental attitude and long-term mortality. The development of interventions to foster a positive outlook may improve the personal and public health of the elderly and prolong life.

## Figures and Tables

**Table 1:** Attitude questions from the Zung self-rated depression scale in the Leisure World Cohort Study, 1980s, *N*=13,674.

Attitude statement	No response	None or a little of the time (1)	Some of the time (2)	A good part of the time (3)	Most or all of the time (4)
	*N* (%)	*N* (%)	*N* (%)	*N* (%)	*N* (%)
My mind is as clear as it used to be	104 (0.8%)	233 (1.7%)	823 (6.0%)	3139 (23%)	9375 (69%)
I find it easy to do the things I used to do	156 (1.1%)	969 (7.1%)	3080 (23%)	5214 (38%)	4255 (31%)
I feel hopeful about the future	263 (1.9%)	530 (3.9%)	1438 (11%)	3170 (23%)	8273 (61%)
I find it easy to make decisions	124 (0.9%)	444 (3.2%)	1596 (12%)	4198 (31%)	7312 (53%)
I feel that I am useful and needed	178 (1.3%)	594 (4.3%)	1573 (12%)	2895 (21%)	8434 (62%)
My life is pretty full	157 (1.1%)	540 (3.9%)	1321 (9.7%)	3286 (24%)	8370 (61%)
I still enjoy the things I used to	120 (0.9%)	514 (3.8%)	1662 (12%)	3374 (25%)	8004 (59%)
Quarters of total attitude (sum of scores for the above seven items)		Q1 7–20	Q2 21–24	Q3 25–26	Q4 27–28

All persons: those with no response on any of the seven items included in no response category	657 (4.8%)	2646 (19%)	3421 (25%)	2757 (20%)	4193 (31%)
All persons: if no response on any item that response recoded as 1 = “none or a little of the time”		3057 (22%)	3633 (27%)	2791 (20%)	4193 (31%)
All persons: if no response on any item that response recoded as 4 = “most or all of the time”		2789 (20%)	3617 (26%)	2917 (21%)	4357 (32%)

**Table 2:** Baseline characteristics in the Leisure World Cohort Study, 1980s.

Characteristic	Total (*N*=13,674)	Men (*N*=4992)	Women (*N*=8682)
	Mean ± SD		
Age at baseline, years	73.6 ± 7.3	74.3 ± 7.2	73.2 ± 7.4
Age at last follow-up, years	87.6 ± 7.4	86.1 ± 7.1	88.5 ± 7.4
Follow-up years	14.0 ± 8.3	11.7 ± 7.7	15.3 ± 8.3
Body mass index, kg/m^2^	23.5 ± 3.3	24.2 ± 2.9	23.1 ± 3.4
Exercise, hours/day	1.0 ± 1.2	1.1 ± 1.3	0.9 ± 1.1
Alcohol, drinks/day	1.4 ± 1.4	1.6 ± 1.5	1.2 ± 1.2
Caffeine, mg/day	171 ± 168	176 ± 172	168 ± 166
	*N* (%)		
History of disease			
High blood pressure	5335 (39%)	1803 (36%)	3532 (41%)
Angina	1547 (11%)	728 (15%)	819 (9.4%)
Heart attack	1396 (10%)	828 (17%)	568 (6.5%)
Stroke	670 (4.9%)	351 (7.0%)	319 (3.7%)
Cancer	1593 (12%)	467 (9.4%)	1126 (13%)
Diabetes	841 (6.2%)	414 (8.3%)	427 (4.9%)
Rheumatoid arthritis	807 (5.9%)	220 (4.4%)	587 (6.8%)
Cigarette use			
Never	6436 (47%)	1664 (33%)	4772 (55%)
Past	5724 (42%)	2900 (58%)	2824 (33%)
Current	1514 (11%)	428 (8.6%)	1086 (12%)
Total attitude			
Missing response	657 (4.8%)	207 (4.1%)	450 (5.2%)
7–20	2646 (19%)	995 (20%)	1651 (19%)
21–24	3421 (25%)	1286 (26%)	2135 (25%)
25–26	2757 (20%)	1051 (21%)	1706 (20%)
27–28	4193 (31%)	1453 (29%)	2740 (32%)
Alive at follow-up	269 (2.0%)	44 (0.9%)	225 (2.6%)

Abbreviation: SD, standard deviation. *p* < 0.0001 for all differences between men and women except caffeine (*p* < 0.009) and total attitude (*p* < 0.0006).

**Table 3:** Attitude and hazard ratio of death among women: the Leisure World Cohort Study, 1981–2016.

	All subjects (*N*=8682)						Excluding first five years of follow-up (*N*=7828)					
	No. subjects^†^	No. deaths	Model 1^a^		Model 2^b^		No. subjects^†^	No. deaths	Model 1^a^		Model 2^b^	
			HR	95% CI	HR	95% CI			HR	95% CI	HR	95% CI
My mind is as clear as it used to be												
None or little of the time	124	121	1.43	1.20, 1.72	1.28	1.07, 1.54	78	75	1.14	0.91, 1.43	1.02	0.81, 1.29
Some of the time	495	491	1.39	1.27 1.52	1.27	1.16, 1.40	406	402	1.43	1.30, 1.59	1.33	1.20, 1.47
A good part of the time	1937	1901	1.09	1.03, 1.15	1.07	1.01, 1.12	1716	1680	1.09	1.03, 1.15	1.07	1.01, 1.13
Most or all of the time	6048	5866	1.00		1.00		5558	5366	1.00		1.00	

I find it easy to do the things I used to do												
None or little of the time	544	539	1.65	1.51, 1.82	1.39	1.26, 1.53	373	368	1.54	1.38, 1.72	1.30	1.16, 1.46
Some of the time	1920	1898	1.29	1.22, 1.37	1.18	1.11, 1.26	1635	1613	1.30	1.22, 1.38	1.18	1.11, 1.26
A good part of the time	3283	3214	1.03	0.98, 1.09	0.99	0.94, 1.04	3072	3003	1.05	0.99, 1.11	1.01	0.95, 1.06
Most or all of the time	2826	2697	1.00		1.00		2664	2535	1.00		1.00	

I feel hopeful about the future												
None or little of the time	331	328	1.53	1.36, 1.71	1.34	1.20, 1.50	242	239	1.46	1.28, 1.66	1.29	1.13, 1.47
Some of the time	924	911	1.25	1.16, 1.34	1.16	1.08, 1.24	785	772	1.22	1.13, 1.31	1.13	1.05, 1.22
A good part of the time	1973	1941	1.09	1.04, 1.15	1.07	1.02, 1.13	1767	1735	1.08	1.02, 1.14	1.07	1.01, 1.13
Most or all of the time	5275	5099	1.00		1.00		4885	4709	1.00		1.00	

I find it easy to make decisions												
None or little of the time	289	282	1.18	1.05, 1.33	1.10	0.97, 1.24	213	206	1.09	0.95, 1.26	1.04	0.90, 1.19
Some of the time	1101	1080	1.10	1.03, 1.17	1.05	0.99, 1.13	967	946	1.11	1.03, 1.18	1.07	0.99, 1.14
A good part of the time	2698	2642	0.96	0.92, 1.01	0.96	0.91, 1.01	2451	2395	0.97	0.92, 1.02	0.96	0.91, 1.01
Most or all of the time	4509	4370	1.00		1.00		4125	3986	1.00		1.00	

I feel that I am useful and needed												
None or little of the time	412	406	1.28	1.16, 1.42	1.14	1.03, 1.26	305	299	1.21	1.08, 1.36	1.09	0.97, 1.22
Some of the time	1038	1027	1.24	1.16, 1.33	1.16	1.09, 1.24	876	865	1.21	1.13, 1.31	1.14	1.06, 1.23
A good part of the time	1750	1705	1.03	0.97, 1.08	1.02	0.96, 1.08	1581	1536	1.02	0.96, 1.08	1.01	0.95, 1.07
Most or all of the time	5364	5203	1.00		1.00		4976	4815	1.00		1.00	

My life is pretty full												
None or little of the time	316	310	1.58	1.41, 1.78	1.33	1.19, 1.50	228	222	1.45	1.26, 1.66	1.22	1.06, 1.40
Some of the time	856	848	1.38	1.28, 1.48	1.25	1.16, 1.35	701	693	1.33	1.23, 1.44	1.21	1.12, 1.31
A good part of the time	1996	1955	1.14	1.08, 1.20	1.09	1.03, 1.14	1788	1747	1.14	1.08, 1.20	1.09	1.03, 1.15
Most or all of the time	5412	5242	1.00		1.00		5034	4864	1.00		1.00	

I still enjoy the things I used to												
None or little of the time	289	285	1.62	1.44, 1.82	1.39	1.23, 1.57	200	196	1.44	1.25, 1.67	1.27	1.10, 1.46
Some of the time	990	973	1.29	1.20, 1.38	1.18	1.10, 1.26	820	803	1.27	1.18, 1.37	1.17	1.08, 1.26
A good part of the time	2011	1978	1.07	1.02, 1.13	1.02	0.97, 1.08	1812	1779	1.07	1.01, 1.13	1.02	0.97, 1.08
Most or all of the time	5304	5133	1.00		1.00		4925	4754	1.00		1.00	

Attitude score (persons with missing items excluded)												
<21	1651	1632	1.38	1.29, 1.46	1.24	1.16, 1.32	1354	1335	1.35	1.26, 1.44	1.22	1.14, 1.31
21–24	2135	2092	1.12	1.05, 1.18	1.07	1.01, 1.13	1927	1883	1.11	1.05, 1.18	1.06	1.00, 1.13
25-26	1706	1662	1.02	0.96, 1.09	0.99	0.94, 1.06	1589	1543	1.03	0.96, 1.10	1.00	0.94, 1.07
27-28	2740	2625	1.00		1.00		2592	2472	1.00		1.00	

Attitude score (missing items given score of 1)												
<21	1925	1904	1.37	1.29, 1.45	1.23	1.16, 1.31	1561	1540	1.34	1.26, 1.43	1.22	1.14, 1.30
21–24	2289	2244	1.12	1.06, 1.19	1.06	1.00, 1.13	2067	2021	1.12	1.06, 1.19	1.07	1.01, 1.13
25-26	1728	1684	1.02	0.96, 1.09	1.00	0.94, 1.06	1608	1562	1.03	0.97, 1.10	1.00	0.94, 1.07
27-28	2740	2625	1.00		1.00		2592	2472	1.00		1.00	

Attitude score (missing items given score of 4)												
<21	1741	1721	1.38	1.30, 1.47	1.24	1.17, 1.32	1418	1397	1.34	1.26, 1.44	1.22	1.14, 1.30
21–24	2272	2227	1.12	1.06, 1.18	1.07	1.01, 1.13	2039	1994	1.11	1.05, 1.18	1.07	1.01, 1.13
25-26	1823	1779	1.04	0.98, 1.10	1.01	0.95, 1.07	1686	1642	1.04	0.97, 1.10	1.01	0.95, 1.08
27-28	2846	2730	1.00		1.00		2686	2570	1.00		1.00	

Abbreviations: CI, confidence interval; HR, hazard ratio. ^†^Subjects do not always total 8682 or 7828 due to those with missing values. ^a^Model 1: adjusted for age (i.e., age as time scale). ^b^Model 2: adjusted for age, smoking, body mass index, exercise, alcohol intake, caffeine consumption, and histories of hypertension, angina, heart attack, stroke, diabetes, rheumatoid arthritis, and cancer.

**Table 4:** Attitude and hazard ratio of death among men: the Leisure World Cohort Study, 1981–2016.

	All subjects (*N*=4992)						Excluding first five years of follow-up (*N*=3980)					
	No. subjects^†^	No. deaths	Model 1^a^		Model 2^b^		No. subjects^†^	No. deaths	Model 1^a^		Model 2^b^	
			HR	95% CI	HR	95% CI			HR	95% CI	HR	95% CI
My mind is as clear as it used to be												
None or little of the time	109	109	1.55	1.27, 1.88	1.41	1.16, 1.71	55	55	1.38	1.06, 1.80	1.34	1.02, 1.75
Some of the time	328	327	1.47	1.31, 1.65	1.31	1.17, 1.47	203	202	1.44	1.25, 1.66	1.30	1.13, 1.50
A good part of the time	1202	1194	1.10	1.03, 1.17	1.06	0.99, 1.13	957	949	1.16	1.08, 1.25	1.13	1.05, 1.21
Most or all of the time	3327	3292	1.00		1.00		2749	2713	1.00		1.00	

I find it easy to do the things I used to do												
None or little of the time	425	425	2.02	1.81, 2.25	1.64	1.46, 1.83	205	205	1.76	1.52, 2.05	1.49	1.28, 1.73
Some of the time	1160	1157	1.32	1.22, 1.42	1.19	1.10, 1.28	848	845	1.32	1.21, 1.44	1.20	1.10, 1.32
A good part of the time	1931	1914	1.10	1.03, 1.18	1.05	0.98, 1.13	1645	1628	1.13	1.05, 1.22	1.09	1.01, 1.17
Most or all of the time	1429	1405	1.00		1.00		1250	1225	1.00		1.00	

I feel hopeful about the future												
None or little of the time	199	199	1.79	1.55, 2.07	1.51	1.31, 1.75	104	104	1.62	1.33, 1.98	1.46	1.20, 1.79
Some of the time	514	512	1.31	1.19, 1.44	1.17	1.06, 1.29	353	351	1.23	1.10, 1.38	1.11	0.99, 1.24
A good part of the time	1197	1188	1.04	0.98, 1.12	0.99	0.92, 1.06	953	944	1.04	0.96, 1.12	0.99	0.92, 1.07
Most or all of the time	2998	2966	1.00		1.00		2520	2487	1.00		1.00	

I find it easy to make decisions												
None or little of the time	155	155	1.90	1.62, 2.24	1.53	1.30, 1.80	71	71	1.83	1.44, 2.31	1.48	1.17, 1.89
Some of the time	495	494	1.17	1.07, 1.29	1.10	1.00, 1.21	354	353	1.14	1.02, 1.27	1.07	0.96, 1.20
A good part of the time	1500	1489	1.07	1.01, 1.14	1.03	0.96, 1.09	1207	1196	1.09	1.01, 1.17	1.04	0.97, 1.12
Most or all of the time	2803	2771	1.00		1.00		2317	2284	1.00		1.00	

I feel that I am useful and needed												
None or little of the time	182	181	1.83	1.57, 2.12	1.64	1.40, 1.90	88	87	1.53	1.23, 1.89	1.44	1.16, 1.78
Some of the time	535	531	1.29	1.17, 1.41	1.14	1.04, 1.25	374	370	1.20	1.07, 1.33	1.06	0.95, 1.18
A good part of the time	1145	1136	1.10	1.02, 1.17	1.08	1.01, 1.16	907	898	1.06	0.98, 1.14	1.05	0.97, 1.13
Most or all of the time	3070	3041	1.00		1.00		2575	2546	1.00		1.00	

My life is pretty full												
None or little of the time	224	223	1.89	1.64, 2.16	1.56	1.36, 1.80	111	110	1.59	1.31, 1.92	1.38	1.14, 1.68
Some of the time	465	462	1.37	1.24, 1.51	1.20	1.09, 1.33	306	303	1.25	1.11, 1.41	1.10	0.97, 1.24
A good part of the time	1290	1279	1.14	1.07, 1.22	1.06	0.99, 1.14	1022	1011	1.14	1.06, 1.23	1.07	1.00, 1.15
Most or all of the time	2958	2929	1.00		1.00		2509	2480	1.00		1.00	

I still enjoy the things I used to												
None or little of the time	225	225	2.23	1.95, 2.56	1.76	1.53, 2.03	86	86	1.86	1.50, 2.31	1.45	1.16, 1.81
Some of the time	672	668	1.36	1.25, 1.48	1.20	1.10, 1.31	455	451	1.31	1.18, 1.45	1.18	1.06, 1.31
A good part of the time	1363	1354	1.11	1.04, 1.19	1.05	0.99, 1.13	1116	1107	1.14	1.06, 1.22	1.08	1.00, 1.16
Most or all of the time	2700	2669	1.00		1.00		2305	2274	1.00		1.00	

Attitude score (persons with missing items excluded)												
<21	995	993	1.54	1.42, 1.67	1.30	1.19, 1.41	631	629	1.43	1.30, 1.58	1.24	1.12, 1.37
21–24	1286	1273	1.16	1.08, 1.25	1.09	1.01, 1.18	1056	1043	1.19	1.10, 1.29	1.13	1.04, 1.23
25-26	1051	1041	1.13	1.04, 1.22	1.06	0.98 1.15	893	883	1.17	1.08, 1.28	1.12	1.02, 1.22
27-28	1453	1435	1.00		1.00		1270	1252	1.00		1.00	

Attitude score (missing items given score of 1)												
<21	1132	1130	1.55	1.43, 1.67	1.32	1.21, 1.43	706	704	1.43	1.31, 1.57	1.25	1.14, 1.38
21–24	1344	1330	1.15	1.07, 1.24	1.08	1.00, 1.16	1103	1089	1.18	1.09, 1.28	1.12	1.03, 1.22
25-26	1063	1053	1.13	1.04, 1.23	1.06	0.98, 1.15	901	891	1.18	1.08, 1.28	1.12	1.03, 1.22
27-28	1453	1435	1.00		1.00		1270	1252	1.00		1.00	

Attitude score (missing items given score of 4)												
<21	1048	1046	1.55	1.43, 1.67	1.31	1.21, 1.42	655	653	1.43	1.30, 1.57	1.24	1.13, 1.37
21–24	1345	1332	1.17	1.09, 1.26	1.10	1.02, 1.18	1092	1079	1.20	1.10, 1.30	1.13	1.04, 1.23
25-26	1094	1084	1.13	1.04, 1.22	1.06	0.98 1.14	924	914	1.17	1.08, 1.28	1.11	1.02, 1.21
27-28	1505	1486	1.00		1.00		1309	1290	1.00		1.00	

Abbreviations: CI, confidence interval; HR, hazard ratio. ^†^Subjects do not always total 4992 or 3980 due to those with missing values. ^a^Model 1: adjusted for age (i.e., age as time scale). ^b^Model 2: adjusted for age, smoking, body mass index, exercise, alcohol intake, caffeine consumption, and histories of hypertension, angina, heart attack, stroke, diabetes, rheumatoid arthritis, and cancer.

**Table 5:** Prospective cohort studies of mental attitude and mortality in the elderly.

First author, date [reference]	Study name Population	Number	Age (years)	Follow-up (years)	Sex	Mental attitude instrument	HR, 95% CI^†^Age- and sex-adjusted Most adjusted model
Gitlay, 2004 [30]	Arnhem Elderly Study The Netherlands	999	65–85	Mean = 9	M,F	Scale of Subjective Well-Being for Older Persons	0.55, 0.42–0.77 0.71, 0.52–0.97
Pitkala, 2004 [32]	Helsinki Aging Study Finland	491	75, 80, 85	10	M,F	6 questions on positive life orientation	0.67, 0.53–0.87^‡^ 0.89, 0.83–0.93
Tindle, 2009 [13]	Women's Health Initiative USA	97,253	50–79	8	F	Life Orientation Test-Revised	— 0.86, 0.79–0.93
Koopmans, 2010 [14]	Arnhem Elderly Study The Netherlands	861	65–85	15	M,F	2 “happiness” questions	0.78, 0.64–0.95 0.92, 0.75–1.14
Benito-Leon, 2010 [29]	Neurological Disorders in Central Spain	2516	65+	7	M,F	Philadelphia Geriatric Center Morale Scale	1.60, 1.29–2.21^‡^ 1.35, 1.00–1.81
Tilvis, 2012 [35]	National Sample Finland	2490	>75	5	M,F	6 questions on positive life orientation	0.65, 0.53–0.81 0.85, 0.67–1.08
Niklasson, 2015 [31]	GERDA Sweden and Finland	646	85+	5	M,F	Philadelphia Geriatric Center Morale Scale	1.73, 1.33–2.26 1.36, 1.03–1.80
St John, 2015 [33]	Manitoba Study of Health and Aging, Canada	1751	65+	5	M,F	Terrible-Delightful Scale	0.75, 0.66–0.86 0.90, 0.78–1.04
Steptoe, 2015 [34]	English Longitudinal Study of Aging	9050	Mean = 65	Mean = 8	M,F	Eudemonic well-being	0.42, 0.36–0.49 0.70, 0.58–0.83
Anthony, 2016 [28]	Rancho Bernardo Study USA	876	50+	< = 12 mean = 8	M,F	Life Orientation Test-Revised	0.98, 0.94–1.02 0.99, 0.94–1.03
Gong, 2016 [10]	Chinese Longitudinal Healthy Longevity Survey	18,676	80–122	16	M,F	7 items on psychological well-being	— 0.84, 0.79–0.88
Liu, 2016 [12]	Million Women Study United Kingdom	719,671	50–69	10	F	“How often do you feel happy?”	1.29, 1.25–1.33 0.98, 0.94–1.01
Kim, 2017 [11]	Nurses' Health Study USA	70,021	Mean = 70	6	F	Life Orientation Test-Revised	0.71, 0.66–0.76^∗^ 0.91, 0.85–0.97
Okely, 2018 [15]	Survey of Health Aging and Retirement in Europe	13,596	50+	∼12	M,F	CASP-12	0.41, 0.38–0.43 0.57, 0.53–0.61

^†^HR for high vs. low category of positive mental attitude, except for Benito-Leon, Niklasson, and Liu studies which compared low vs. high category. ^‡^Unadjusted HR. ^∗^Adjusted for additional demographic factors as well.

## Data Availability

The data used to support the findings of this study are available from the corresponding author upon request.

## Abbreviations

BMI: Body mass index
CI: Confidence interval
HR: Hazard ratio
PGCMS: Philadelphia Geriatric Center Morale Scale
LOT-R: Life Orientation Test-Revised
RR: Relative risk.
